# And so we begin: Introducing the *Annals of the Child Neurology Society*

**DOI:** 10.1002/cns3.1

**Published:** 2022-12-22

**Authors:** E. Steve Roach, Phillip L. Pearl

**Affiliations:** ^1^ The University of Texas Dell Medical School Austin Texas USA; ^2^ Harvard Medical School Boston Massachusetts USA

*Knowledge is of two kinds: we know a subject ourselves, or we know where we can find information upon it*.

Samuel Johnson

This editorial marks the formal beginning of the *Annals of the Child Neurology Society* (ACNS), an official journal of the Child Neurology Society (CNS). Since its founding in 1972, the CNS membership has increased steadily and the needs of its members have become more diverse. This is an extraordinarily productive era for the study of childhood neurological disorders, and the steady stream of exciting discoveries make this an ideal time for the society to launch its clinically focused journal.

The CNS will maintain its traditional relationship with *Annals of Neurology*, with its focus on more basic research. Several years ago, the American Neurological Association created *Annals of Clinical and Translational Neurology*, and the addition of ACNS by the CNS forms an *Annals* “family” of journals that together support a wide range of scholarly endeavors.

Creation of ACNS is arguably the most important venture for the society in many years. All new journals face challenges both expected and unexpected, and the fledgling journal will need time to grow and mature. But the CNS needs a clinically focused journal, and we will succeed.

## What is the Scope of ACNS?

ACNS provides a venue for clinically focused articles and for society business. We will publish clinical and translational research articles, epidemiology studies, case series, case reports, educational image vignettes, quality improvement articles, letters, and commentaries on medicine or societal factors that affect the care of children with neurological disease. Clinically relevant basic science articles are encouraged. Manuscripts must undergo rigorous peer review and revision before acceptance.

## Introducing the Editorial Team

We have assembled an outstanding editorial team (Table 1) and a diverse editorial board whose members have broad expertise in child neurology as well as in important areas such as neuroradiology and neurosurgery (Table 2). The editorial board includes both established leaders in the field and up‐and‐coming colleagues who represent the future of the profession. While centered in North America, ACNS has editorial board representatives from Africa, South America, Asia, Europe, and the Middle East.

**Table 1:** *Annals of the Child Neurology Society* associate editors.

Phillip Pearl	Senior associate editor
Boston, MA	
David Bearden	Infectious disease
Rochester, NY	
Dave Clarke	Epilepsy
Austin, TX	
Bruce Cohen	Medical economics and practice
Akron, OH	
Janet Soul	Fetal and neonatal neurology
Boston, MA	
Yingchao Yuan	Statistics & epidemiology
Austin, TX	
John Crawford	Neuro‐oncology
Orange, CA	
Donald Gilbert	Movement disorders
Cincinnati, OH	
Partha Gosh	Neuromuscular disorders
Boston, MA	
Kristina Julich	Genetic disorders
Austin, TX	
Ann Neumeyer	Behavioral disorders
Boston, MA	
Grace Gombolay	Neuroimmunology
Atlanta, GA	

**Table 2:** *Annals of the Child Neurology Society* editorial board members.

Gyula Acsadi, Farmington, CT
Stephen Ashwal, Loma Linda, CA
Erica Augustine, Baltimore, MD
Elizabeth Berry‐Kravis, Chicago, IL
Lauren Beslow, Philadelphia, PA
Heidi Blume, Seattle, WA
Josh Bonkowsky, Salt Lake City, UT
James Nickolas Brenton, Charlottesville, VA
Audrey Brumback, Austin, TX
Juan Cabello, Valparaiso, Chile
Carol Camfield, Halifax, NS
Peter Camfield, Halifax, NS
Steven Chrzanowski, Boston, MA
Melissa Chung, Columbus, OH
Gary Clark, Houston, TX
Anne Comi, Baltimore, MD
Dana Cummings, Pittsburgh, PA
Jay Desai, Los Angeles, CA
Francis DiMario, Hartford, CT
Marc DiSabella, Washington, DC
William Dobyns, Minneapolis, MN
Kevin Ess, Nashville, TN
Laura Flores‐Sarnat, Calgary, AB
David Franz, Cincinnati, OH
Suman Ghosh, Gainesville, FL
Tanjala Gipson, Memphis, TN
Meredith Golomb, Indianapolis, IN
Howard Goodkin, Charlottesville, VA
Rejean Guerriero, St. Louis, MO
Christina Gurnett, St. Louis, MO
Charles Hammond, Kumasi, Ashanti, Ghana
Duriel Hardy, Austin, TX
Franziska Hoche, Boston, MA
Sarah Hopkins, Philadelphia, PA
Monica Islam, Columbus, OH
Charuta Joshi, Dallas, TX
Sergiusz Jozwiak, Warsaw, Poland
Csaba Juhasz, Detroit, MI
Louisa Kalsner, Hartford, CT
Matthew Kirschen, Philadelphia, PA
Eric Kossoff, Baltimore, MD
Wang Tso Lee, Taipei, Taiwan
Jean‐Baptiste Le Pichon, Kansas City, MO
Monica Lemmon, Durham, NC
Kenneth Mack, Rochester, MN
David Mandelbaum, Providence, RI
Joseph Madsen, Boston, MA
Soe Mar, St. Louis, MO
Jonathan Mink, Rochester, NY
Solomon Moshe, New York, NY
Finbar O′Callaghan, London, UK
Roger Packer, Washington, DC
Steven Pavlakis, New York, NY
Anna Pinto, Boston, MA
Nancy Rollins, Dallas, TX
Sean Rose, Columbus, OH
Harvey Sarnat, Calgary, AB
Renee Shellhaas, Ann Arbor, MI
Michael Shevell, Montreal, QC
Harvey Singer, Baltimore, MD
Steven Sparagana, Dallas, TX
Jonathan Strober, San Francisco, CA
Lisa Sun, Baltimore, MD
Yoshiyuki Suzuki, Tokyo, Japan
Haluk Topaloglu, Istanbul, Turkey
William Trescher, Hershey, PA
Hannah Tully, Seattle, WA
Elizabeth Tyler‐Kabara, Austin, TX
Jithangi Wanigasinghe, Colombo, Sri Lanka
Elaine Wirrell, Rochester, MN
Max Wiznitzer, Cleveland, OH

## Oversight of ACNS

ACNS is owned by the CNS and will be published via a contract with Wiley, much like the arrangement for *Annals of Neurology* between the American Neurological Association and Wiley. The society maintains editorial control of ACNS, selecting the publishing partner, choosing the editor‐in‐chief, and approving associate editors and editorial board members. At least 65% of the editorial board members must be CNS members in good standing. The ACNS editorial team will make the final determination of a manuscript′s suitability for publication.

## Why an Open Access Journal?

The articles in open access journals are freely available to anyone with internet access and, consequently, reach a larger audience and tend to be cited more often than equivalent articles in subscription journals. Both open access and subscription‐based journals, however, have production costs. Medical journals have for many years been funded by subscription fees and product advertising. The positive aspect of this funding method is that the authors are not asked to share the cost of publication. On the down side, these journals are only available to individuals with an individual subscription or access to a medical library.

The landscape for funding medical journals has shifted dramatically in recent years. Most readers now read articles online after searching for information about a specific topic. With little opportunity for effective ad placement, the revenue from medical journal advertising has plummeted. More and more journals are reducing expenses by eliminating their costly and environmentally unsound print editions. Some subscription journals have adopted a hybrid model, mixing open access articles whose authors pay a publication fee and traditional restricted access articles with no publication fee.

Some authors may have difficulty paying open access fees, so despite the trend toward open access journals, there will continue to be a role for subscription‐based journals. We have developed a number of ways to mitigate the publication fees. CNS members will receive a 20% discount on reviews and research articles. The society will pay the fees for articles whose first author is a junior CNS member. They will ask that award lectures and society‐supported symposium presentations be followed by a companion manuscript for ACNS and will pay the costs of these articles. Wiley allows free or reduced publication fees for articles from authors living in countries with limited resources.

## Contacting ACNS

Preliminary inquiries about the suitability of topics or other queries should be directed to the editor‐in‐chief, Dr. E. Steve Roach, at roache@austin.utexas.edu or to the appropriate associate editor via AnnalsCNS@austin.utexas.edu. For information about manuscript preparation, submission, or publication costs, please visit the ACNS website at https://www.annalscns.com. Follow ACNS on Twitter (@AnnalsCNS).

